# Mechanism for epeirogenic uplift of the Archean Dharwar craton, southern India as evidenced by orthogonal seismic reflection profiles

**DOI:** 10.1038/s41598-021-80965-7

**Published:** 2021-01-15

**Authors:** Biswajit Mandal, V. Vijaya Rao, P. Karuppannan, K. Laxminarayana

**Affiliations:** CSIR-National Geophysical Research Institute, Hyderabad, 500007 India

**Keywords:** Environmental sciences, Solid Earth sciences

## Abstract

Plateaus, located far away from the plate boundaries, play an important role in understanding the deep-rooted geological processes responsible for the epeirogenic uplift and dynamics of the plate interior. The Karnataka plateau located in the Dharwar craton, southern India, is a classic example for the plateau uplift. It is explored using orthogonal deep crustal seismic reflection studies, and a mechanism for the epeirogenic uplift is suggested. A pseudo three-dimensional crustal structure derived from these studies suggests a regionally extensive 10 km thick magmatic underplating in the region. It is further constrained from active-source refraction and passive-source seismological data. We interpret the Marion and Reunion mantle plume activities during 88 Ma and 65 Ma on the western part of Dharwar craton are responsible for the magmatic underplating, which caused epeirogenic uplift. Flexural isostasy related to the onshore denudational unloading and offshore sediment loading is also responsible for the persisting uplift in the region. Plate boundary forces are found to be contributing to the plateau uplift. The present study provides a relationship between the mantle plumes, rifting, development of continental margins, plateau uplift, and denudational isostasy. Combination of exogenic and endogenic processes are responsible for the plateau uplift in the region.

## Introduction

Plateaus are broad uplands of considerable elevation and occur on the continents (e.g., Colorado) as well as on the ocean floor (e.g., Iceland, Hawaii). Plateaus are an integral part of all continents. Some of them are related to convergent and divergent plate margins, and others are far away from these margins (e.g., Tibet and Shillong-convergent, Ethiopia-divergent, Colorado-intraplate). Plateau uplifts, especially those away from the plate margins, provide important inputs to understand the interplate geo-dynamics because of the involvement of deep-rooted geological processes that are different from the active subduction environment.

Passive continental margins are evolved due to active rifting and extensional tectonics. These margins, world over, are characterized by huge linear escarpments, which separate a lower-elevation coast-parallel plain from an elevated (low relief) inland plateau. Marginal uplifts and the presence of elevated regions (plateaus) adjoining passive rifts are common geological phenomenon along recent continental margins. However, the precise mechanism for these uplifts remains debatable. Important among the possible mechanisms responsible for plateau uplift are physical thickening of crust, thermal expansion and thinning of the lithosphere^1,2^, phase change in the lithosphere (basalt-eclogite, spinel-olivine), delamination of the mantle lithosphere^3^, magmatic underplating^4^ and flexural response to denudation^5,6^. Some of these processes occur during rifting and operate only for a short period and thereby it can’t explain the post-rift uplift^7^. The passive continental margins remain elevated and continue to rise over geological times. Gunnell^8^ with a comprehensive review divides the underlying principles of plateau uplift broadly into three categories: isostasy—isostatic response to the reduction in density either due to mechanical or thermal processes (exogenic and endogenic), crustal buoyancy—increase in lithospheric thickness, and lithospheric flexure—plastic necking due to lithospheric stretching or asymmetric denudation on either side of the scarp.

**Figure 1:**
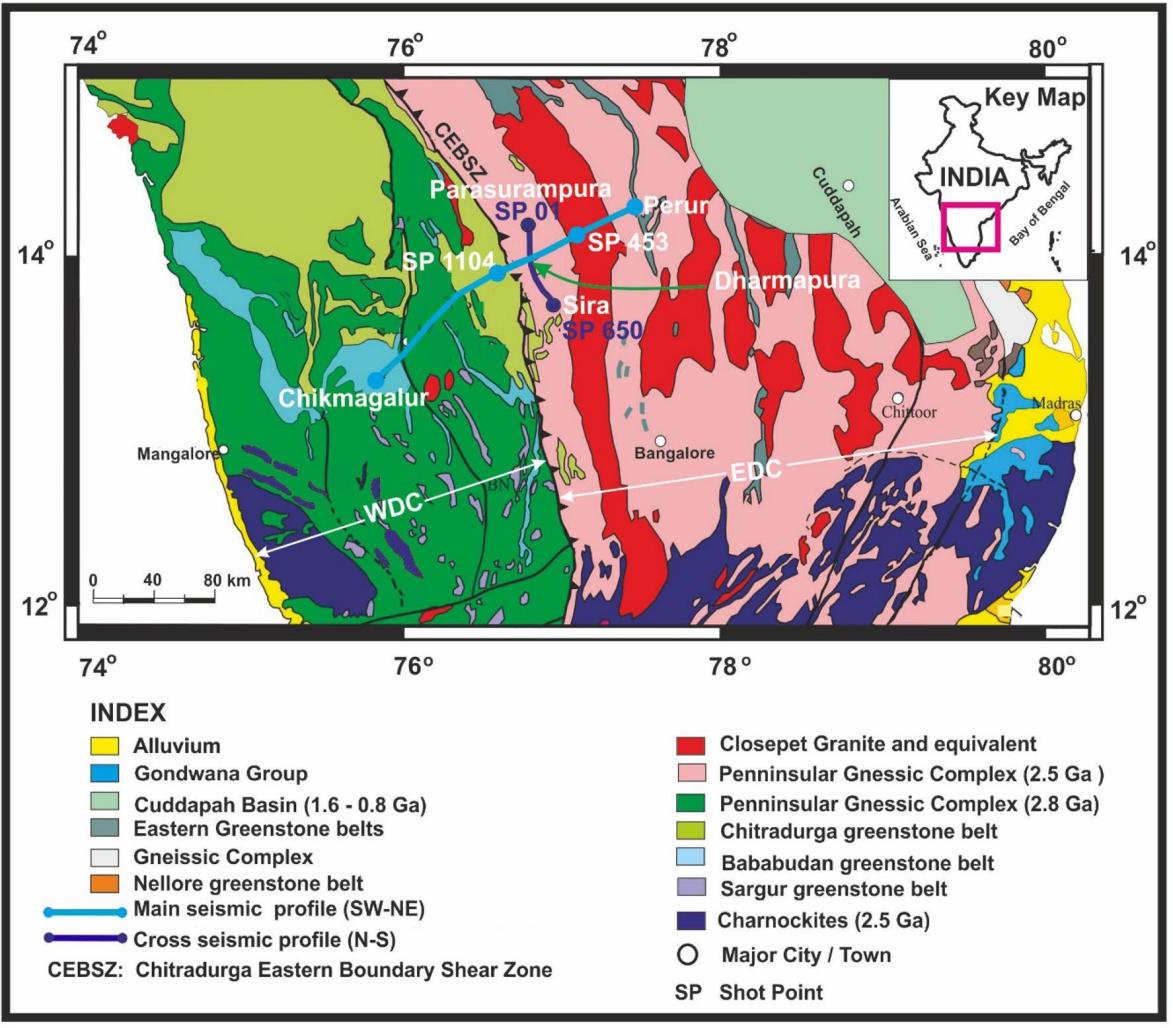
Geological map of Archean Dharwar craton, southern India, along with the Perur-Chikmagalur main and Parasurampura-Sira cross-profiles marked over it. (modified after Vijaya Rao et al.^11^). EDC-Eastern Dharwar Craton; WDC-Western Dharwar Craton; CEBSZ-Chitradurga Eastern Boundary Shear Zone.

**Figure 2:**
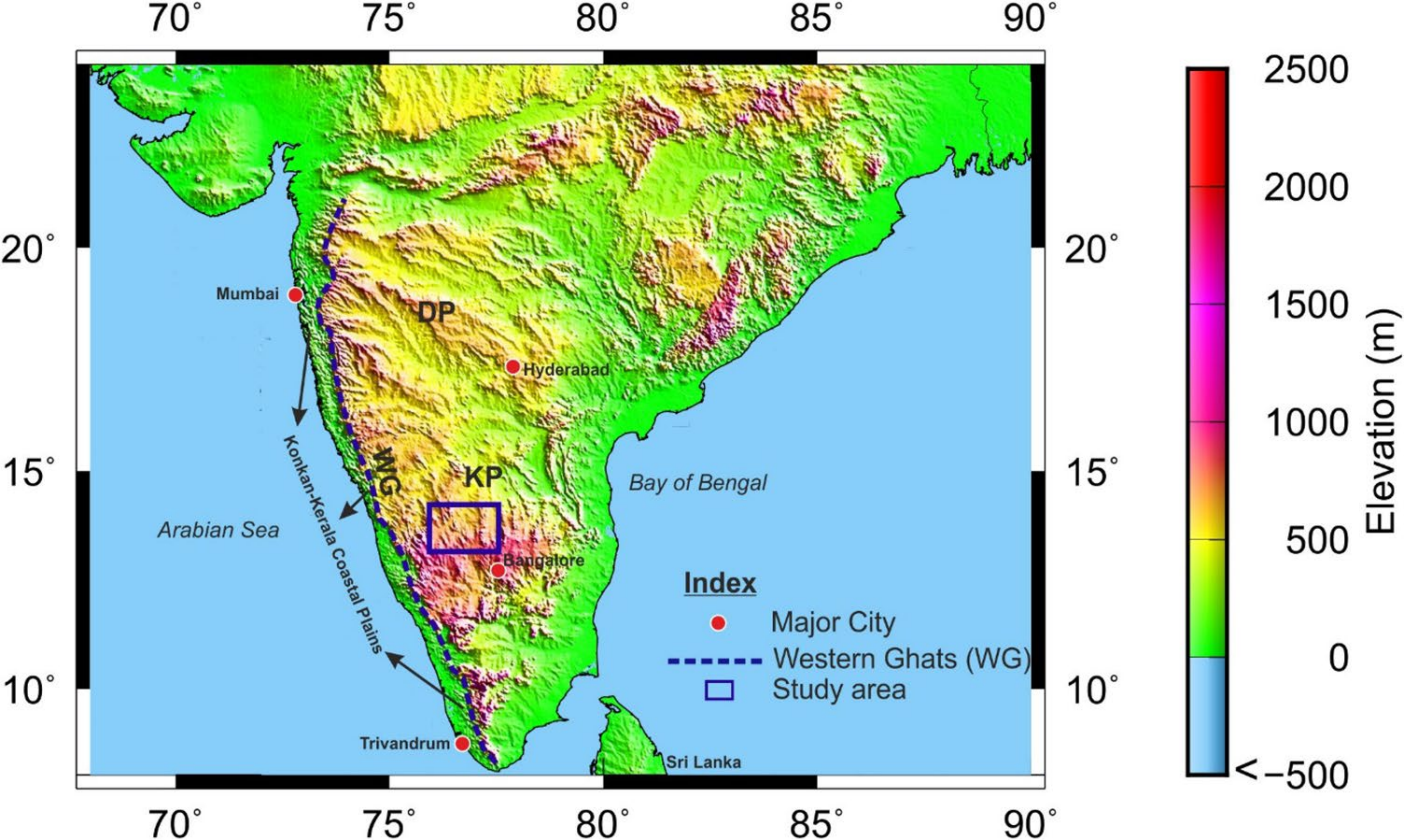
The elevation map of the Karnataka plateau and adjoining regions of southern India plotted using Generic Mapping Tools (GMT) (after Wessel et al.^12^), a free software. Elevation data (after Smith et al.^13^) is downloaded from https://topex.ucsd.edu/cgi-bin/get_data.cgi. N–S dashed line represents the Western Ghats (WG). KP Karnataka Plateau, DP Deccan Plateau.

The major part of peninsular India represents a plateau with an average elevation of around 500 m^9^. To the west of the plateau lies a 1500 km long Western Ghats escarpment with elevations varying from 2400 to 400 m. The western part of the Western Ghats is a low-lying 50 km wide Konkan-Kerala coastal plain. The present study region (Fig. 1) is a part of the Archean Dharwar craton and also an uplifted region referred as the Karnataka plateau (Fig. 2, KP). This plateau, a part of the elevated region, is contiguous with the Deccan plateau (Fig. 2, DP) located to its north, which together occupies an area of more than 400,000 sq. km^10^.

The Karnataka plateau is covered with Meso-Neoarchean gneisses and greenstone belts, whereas the Deccan plateau is covered with late Cretaceous (65 Ma) Deccan volcanics. Various mechanisms (cited earlier) were attributed to the plateau uplift in the region. But, there were no convincing pieces of evidence from the subsurface structural details. In the present study, orthogonal seismic reflection profiles are used to understand and suggest a mechanism of epeirogenic uplift of the Karnataka Plateau and the age of its formation.

Seismic reflection studies provide great details regarding the structure and tectonic evolution of the continental crust. They are used to understand the crustal structure by traversing a profile orthogonal to the strike, thereby determining the dip of the reflector. In areas where crustal structure exhibits unpredictable three-dimensional (3-D) geometry, the two dimensional (2-D) seismic profile cannot provide the appropriate structure. In such areas, the crustal structure is accurately mapped by 3-D techniques. Even though a network of 2-D profiles or 3-D (areal) crustal seismic studies are appropriate, they are prohibitively costly. Alternatively, seismic data can be acquired in long linear main profiles, accompanied by smaller cross-profiles for limited control in the directions away from the main profile. Such a field configuration is more suitable for reconnaissance surveys of 3-D crustal structure, which will be helpful to understand the geodynamics of the region.

The 3D structure also provides the opportunity to understand the relationship between profile direction and strike/dip. Seismic studies with such field configuration were carried out in the Dharwar craton to understand the broad regional structure features. Similar studies are being carried out in several areas, such as the Canadian shield^14^, the Cordillera^15^, across the Eastern Alps^16^, and NW Scotland using BIRPS data^17^.

### Tectonic framework

Indian shield is a mosaic of several Archean cratonic blocks, including the Dharwar craton, and sutured together with Proterozoic mobile belts between them. The Archean Dharwar craton is one of the oldest and largest Archean cratonic blocks of the world. It is a classic granite-greenstone terrain with a 3.5 Ga geological history^18^. The Dharwar craton is made-up of the Mesoarchean Western (WDC) and Neoarchean Eastern Dharwar Cratons (EDC). There are differences of opinion regarding the tectonic evolution and the location of the suture zone between the WDC and EDC.

**Figure 3:**
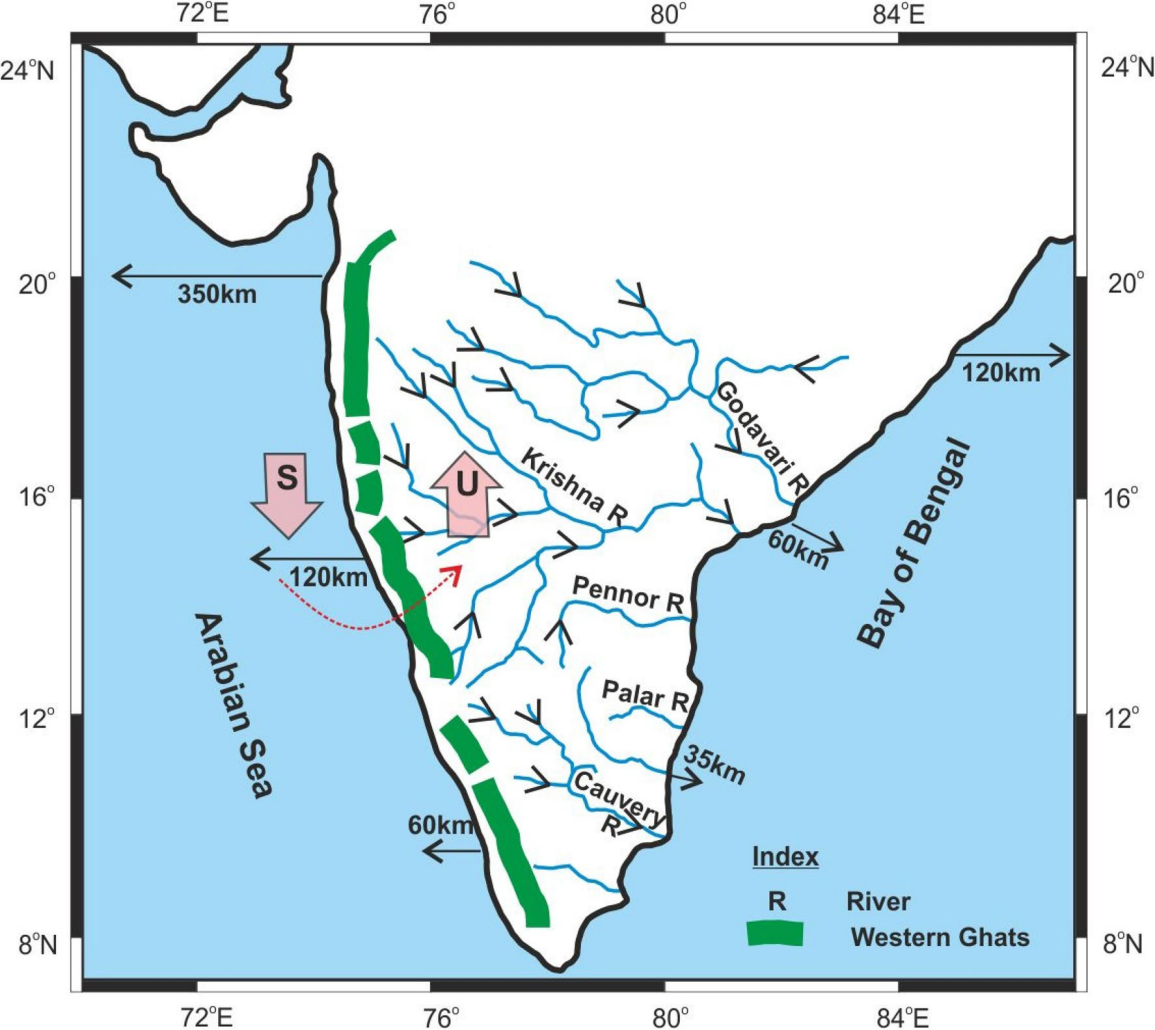
Drainage pattern of peninsular India developed due to mantle plume activity shows asymmetric relief with eastward tilting from 1.5 km high Western Ghats escarpment toward the flood plains of eastward-flowing rivers. Arrows from the coast indicate the width of the continental shelf. The shelf area decreases from north to south and has an area of about 310,000 sq. km^19^ in the west and 2493 km long shoreline in the east. S-Subsidence, U-uplift. Onshore denudational unloading and offshore sedimentary loading lead to subsidence (S) of the offshore continental margin. Such a huge redistribution of crustal loads leads to onshore uplift (U) due to upward flexure of the lithosphere (effect of isostatic compensation) because of rotation (shown as red colour dashed arrow) due to some form of mechanical coupling between the offshore and onshore regions. (Map is modified after Radhakrishna^9^).

India was a part of the Gondwana Supercontinent during the Phanerozoic. The Gondwana supercontinent brokeup during the Mesozoic. During this process, Madagascar and Seychelles separated from India during 88 Ma and 65 Ma, respectively, due to the Marion and Reunion mantle plume activities. It has generated a passive western continental margin and Arabian sea. It has also developed asymmetric topography manifested by Western Ghats escarpment to the west and eastward draining river pattern^1^ (Fig. 3). Similarly, the separation of India from Australia and Antarctica during the Cretaceous (~ 130 Ma) has generated the eastern continental margin and the Indian Ocean (Bay of Bengal). The eastern and western continental margins developed a huge shelf area (Fig. 3) with a thick sedimentary pile due to the drainage pattern^19^. The westerly drainage consists of short rivers emanating from the Western Ghats. The Western continental margin of India is geomorphologically similar to other rifted provinces like the Parana of Brazil, Karoo of SE Africa, and Etendeka of SW Africa^1,6^.

### Seismic study

A DHARSEIS experiment was conducted to understand the structure, dynamics, and tectonic evolution of the Dharwar craton and to delineate the accretionary boundary between the WDC and EDC^11^. Further, it is designed to understand the mechanism of the plateau uplift in the region. It includes a 200 km long coincident deep seismic reflection and refraction/wide-angle reflection study in the NE-SW trending Perur-Chikmagalur main profile and a 66 km long reflection study along the Parasurampura-Sira N-S cross-profile, orthogonal to main profile (Fig. 1). Seismic data were acquired during 2010–2012. The main profile was recorded across the strike, whereas the cross-profile was recorded along the strike. Elevation along the main profile varies between 1000 to 600 m and ~ 600 m along the cross profile. The seismic experiment was designed to obtain 3-D information on subsurface crustal structure across a gneissic terrain nearer to the Neoarchean suture zone.

Deep crustal seismic studies along the main profile provided the subsurface crustal structure and velocity-depth model^11,20,21^. These studies suggested accretion of the WDC and EDC during the Neoarchean convergence based on the differences in crustal structure, the Moho geometry, and crustal thickness between them. During this orogenic process, the EDC was subducted below the WDC with a mantle suture at the eastern part of the Closepet granite (Fig. 1). The eastern boundary of the Chitradurga greenstone belt is identified as the surface expression of the suture zone and referred to the Chitradurga Eastern Boundary Shear Zone (CEBSZ). Lack of 3-D control was hindering proper understanding of the tectonic evolution of the Dharwar craton. The cross-profile may fill the gap to some extent.

During the present study, we processed the seismic reflection data from the cross-profile using the Common Reflection Surface (CRS) stack approach. We then compare these results from that of the main-profile, which was previously published by Mandal et al. in 2018^21^. The objective of the present paper is to derive a pseudo-3-D seismic image of the study area, which can be utilized to understand the implications of the plateau uplift of the region and to identify the role of profile direction on the seismic section. The present study is the first deep seismic reflection study to understand the 3-D crustal structure of the Dharwar craton.

## Seismic reflection data

### Data acquisition

Crustal seismic reflection data were acquired along a 200 km long Perur-Chikmagalur main profile (Fig. 1) with an end-on field geometry using a 150-channel EAGLE-88 Radio-Frequency-telemetry acquisition system. Shots and receivers’ intervals were kept at 200/100 m and 100 m, respectively. A charge size of 50–75 kg explosives was loaded with specially drilled shot holes to a depth of 25–28 m that is used as a seismic source. The data were acquired using ten 4.5 Hz geophones-string. It is recorded up to a length of 24 s with a 4 ms sampling interval.

Crustal seismic reflection data were also acquired along a 66-km long N-S trending Parasurampura-Sira cross-profile (Fig. 1) with asymmetric split-spread geometry (12 + 6 km) using a 180-channel SCORPION cable-telemetry system. Shots and receivers’ intervals were kept at 200 m and 100 m, respectively. Explosives were used as a source similar to that of the main profile. The data were acquired using ten 10 Hz geophones-string with a sample interval of 2 ms and 24 s record length. More details of seismic reflection data acquisition from both profiles are shown in Table 1. Both datasets were acquired independently.

**Table 1:** Data acquisition parameters for the main and cross profiles.

Parameters	Main profile (Perur-Chikmagalur)	Cross profile (Parasuramura-Sira)
Length of profile	200 km	66 km
Type of source	Explosives	Explosives
Shot hole depth	25–28 m	25–28 m
Charge size/hole	50–75 kg	50–75 kg
No. of shots	900	167
No. of channels	150	180
Shot point spacing	200 m	200 m
Receiver spacing	100 m	100 m
Source-receiver offset	100 m (nearest), 15,000 m (farthest)	100 m (nearest), 12,000 m (farthest)
Spread length	15 km	18 km
Foldage (theoretical)	37	37
Type of spread	End-on	Asymmetric split (12 + 6 km)
Record length	24 s	24 s
Sampling interval	4 ms	2 ms
Type of magnetic tape	IBM 3490 cartridge	LTO Tape
Type of data	SEG-D, Demultiplexed	SEG-Y, Demultiplexed
Frequency range	4.5–250 Hz	10–250 Hz
Uphole recording	Yes	Yes
Instrument used	Eagle-88, RF Telemetry system	Scorpion, Line Telemetry system
Geophones type	4.5 Hz, 10 phone string, Bunching	10 Hz, 10 phone string, Bunching

### Data processing

We processed the seismic reflection data using the CRS approach. The CRS approach is another way of processing Common Mid-Point (CMP) data. It overcomes some of the limitations of the conventional CMP method. This approach considers the seismic reflection data in common reflection surface, instead of common reflection points, thereby more data are included in the stack, and signal to noise ratio (S/N) increases by many folds. In the CRS approach, the data are stacked using three parameters, namely the angle of emergence (α), the radii of curvature of normal incidence point wave (R_NIP_) and normal wave (R_N_)^22–24^, instead of a single parameter, the stacking velocity used in the CMP method. Further, the CRS-parameters do not need a precise velocity model to stack the data, as in the case of the CMP method.

**Figure 4:**
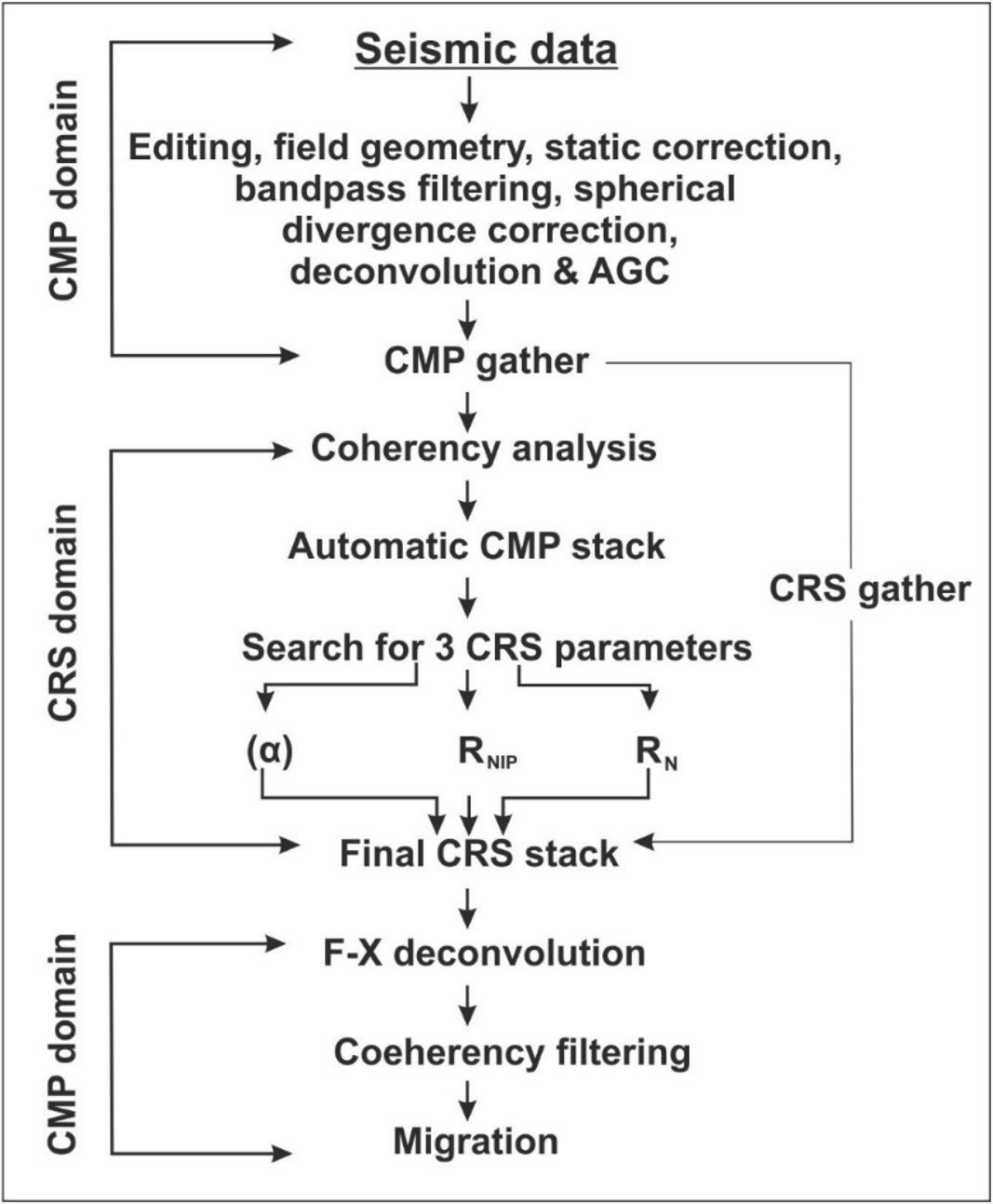
CRS data processing flow chart. α—the angle of emergence, R_NIP_—radius of curvature of normal incidence point wave, and R_N_—radius of curvature of the normal wave.

Processing steps used for cross and main profiles are similar. Most of the processing steps for CMP and CRS approaches are the same, except for the stacking procedure. Again, the post-stack processing steps are the same as that of CMP. The data processing flow chart is shown in Fig. 4.

Initially, all random noises are edited, and reverse polarities of traces are corrected. Next, field geometry is applied. Static correction, bandpass filtering, spherical divergence correction, deconvolution, and automatic gain control (AGC) are applied to the field data. Then, the data are transferred to the CRS domain. In this domain, initially, coherency analysis is carried out, and the best coherency section is selected. It is used to generate an automatic CMP stack section that is a replica of the CMP stack, as found in the standard CMP technique. This automatic CMP stack is used to calculate CRS parameters (α, R_NIP,_ R_N_). Finally, the CRS stack is obtained using these parameters.

**Figure 5:**
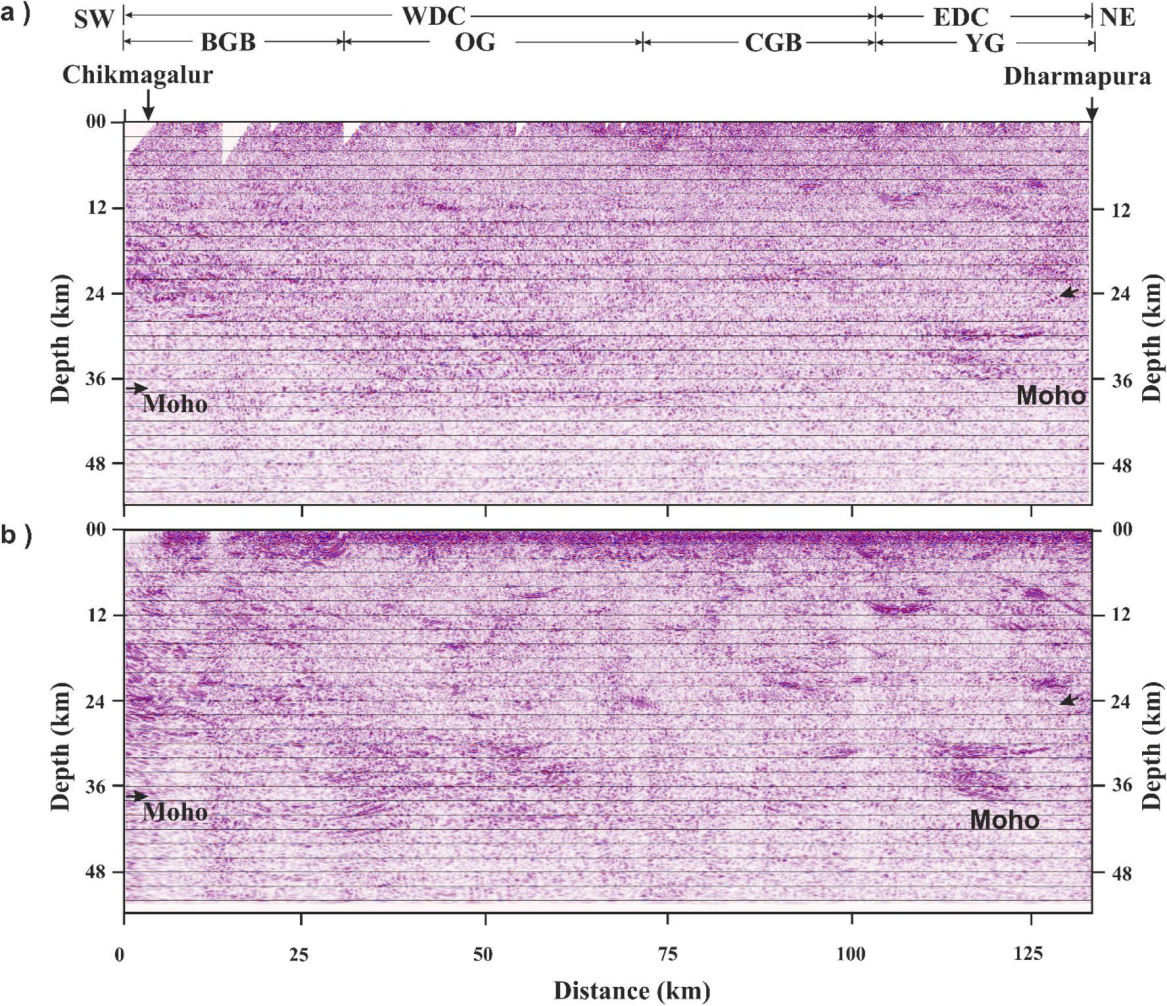
Comparison of conventional CMP and relatively new CRS time-migrated depth sections of the main profile. It shows differences in seismic sections due to differences in processing approach (see text for processing details). (**a**) No prominent reflection bands are observed in the seismic section, (**b**) The Moho is bright and continuous in CRS section (after Mandal et al.^21^). Prominent NE dipping reflection bands are observed at the beginning and end of the seismic section.

**Figure 6:**
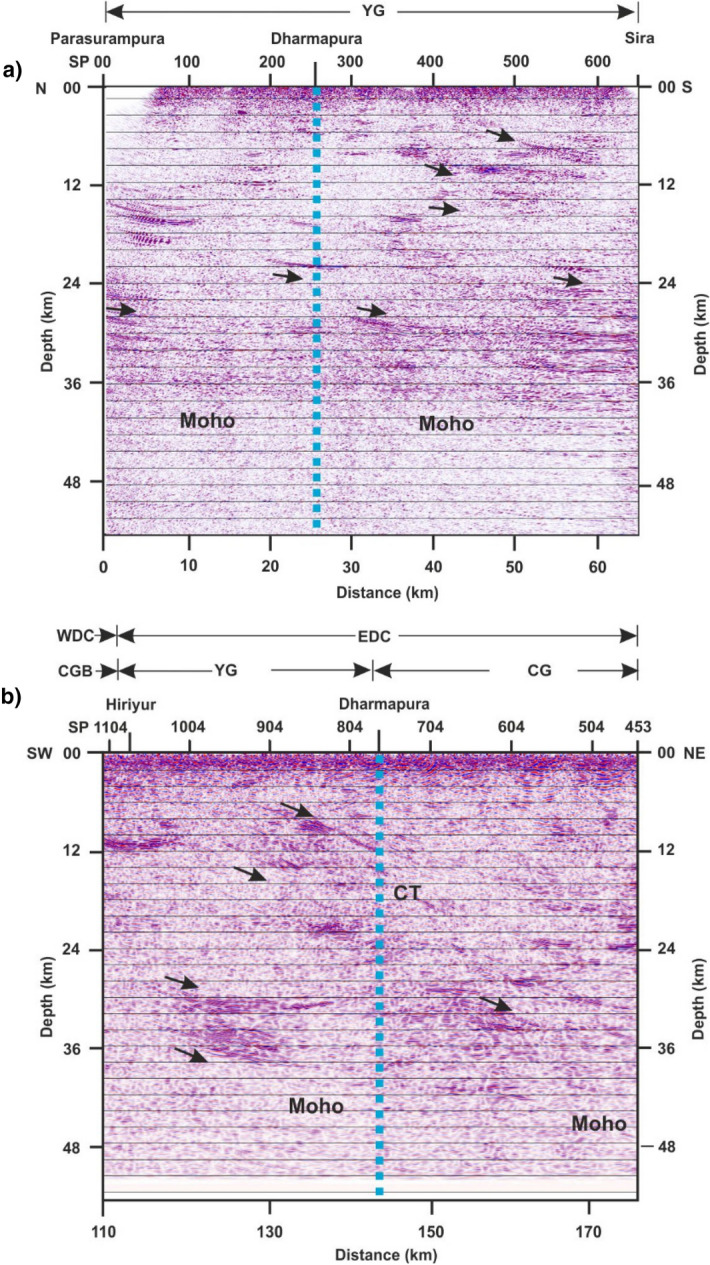
CRS migrated seismic reflection section along (**a**) Parasurampura-Sira cross-profile, along the strike. A subhorizontal reflection band observed from 16–24 km depth corresponds to the Chitradurga thrust of the main profile. (**b**) A part of the main profile (65 km long), across the strike (after Mandal et al.^21^). The vertical dashed line represents the intersection of the two profiles. YG-Younger Granite; CGB-Chitradurga Greenstone Belt; CG-Closepet Granite; EDC-Eastern Dharwar Craton; WDC-Western Dharwar Craton; CT-Chitradurga Thrust. Arrows indicate dipping reflection fabric representing the structural features of the region.

The CRS-stack section is time-migrated and presented in depth using the velocity information from the coincident refraction data^11^. The conventional CMP and relatively new CRS stacking images are presented in Fig. 5a and b for comparison. The superiority of the CRS section over the CMP image is very clear. Depth migrated sections of the cross, and main profiles are presented in Fig. 6a and b to the same length.

### Seismic sections and interpretation

The time-migrated seismic depth section along the cross-profile, imaged in the present study, is presented in Fig. 6a. It shows mostly subhorizontal to gently dipping reflection bands at different depths, extending from 4 to 42 km. The depth of the reflectors is determined using the velocity-depth model of the coincident refraction / wide-angle reflection study along the main profile^11^. The migrated seismic depth section along the main profile^21^, the same length to that of cross-profile, is presented in Fig. 6b. A comparison is made between seismic sections from both the profiles. The main profile shows a dipping reflection fabric extending from 6 to 28 km depth that sole into prominent subhorizontal lower-crustal reflections (Fig. 6b). The above dipping reflection fabric referred to the Chitradurga Thrust (CT) is developed by accommodating the crustal shortening during the Neoarchean convergence, subduction, and accretion of WDC and EDC^11,21^. Contrarily, the same thrust is observed as a subhorizontal reflection band between 16 and 24 km depth in the cross-profile (Fig. 6a).

The thickness of this band is approximately the same as the width of the Chitradurga thrust at the intersection of the profiles. The difference in the crustal structure of a subsurface dipping-reflector between two orthogonal profiles is due to the profile direction with respect to the strike. Thus, the present study demonstrates the role of profile direction relative to the strike.

**Figure 7:**
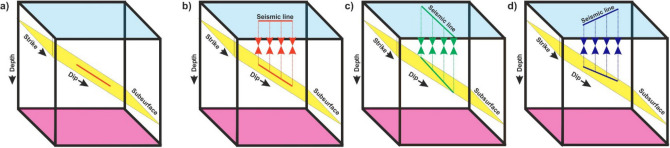
Schematic diagram showing the subsurface structure of a reflector with respect to profile direction. (**a**) Actual structure of the subsurface reflector Structure observed on a profile traversing: (**b**) across the strike-direction (**c**) along strike-direction, (**d**) some arbitrary angle to the strike of the profile.

A subhorizontal lower-crustal reflection band is observed between 30 and 40 km depth both in the cross and main profiles (Fig. 6a,b). It is in contrast to the dipping reflector, which shows different images for the same sub-surface feature depending on the profile direction. The present data demonstrate that the linear features are unchanged, whereas dipping features in a seismic section change as derived from the orthogonal profiles. 3-D crustal reflection studies from different parts of the world, like the BIRPS (British Institution Reflection Profiling Syndicate) and COCORP (COnsortium for COntinental Reflection Profiling) groups^15,16^ observed similar structural patterns across and along the strike, as observed in the present study. The present study well demonstrates, with field examples, the role of profile direction with respect to strike and dip. The image of a subsurface reflector observed in a seismic section varies as per the profile direction with respect to strike, which is illustrated schematically in Fig. 7a–d. The dip of the reflector (Fig. 7a) remains the same in a profile orthogonal to strike (Fig. 7b), whereas it varies as per the direction of the profiles and finally not observed along the strike direction (Fig.7c).

A gently south-dipping reflection fabric is seen from 6 to 12 km depth in the southern part from 30 to 60 km horizontal distance along the cross-profile (Fig. 6a). The Moho along this profile is also gently dipping towards the south, with its depth changing from 40 to 42 km from north to south. In general, most of the reflections dip toward the south. Thus, with this 3-D structural control provided by cross-profile, we conclude that the structural grain of the Dharwar craton dips to the south.

### Epeirogenic uplift

The lower-crustal subhorizontal reflection fabric observed between 30 and 40 km depth (Fig. 5a) represents a transition zone from lower-crustal material to upper mantle material. We interpret the base of this reflection fabric as the Moho. The laminar nature of this reflection band is generated due to the accretion of the upper mantle material at the base of the crust, which is referred to as the magmatic underplating^25,26^. A similar lower-crustal feature is also observed in several regions of the world and interpreted as magmatic underplating^11,27–29^.

**Figure 8:**
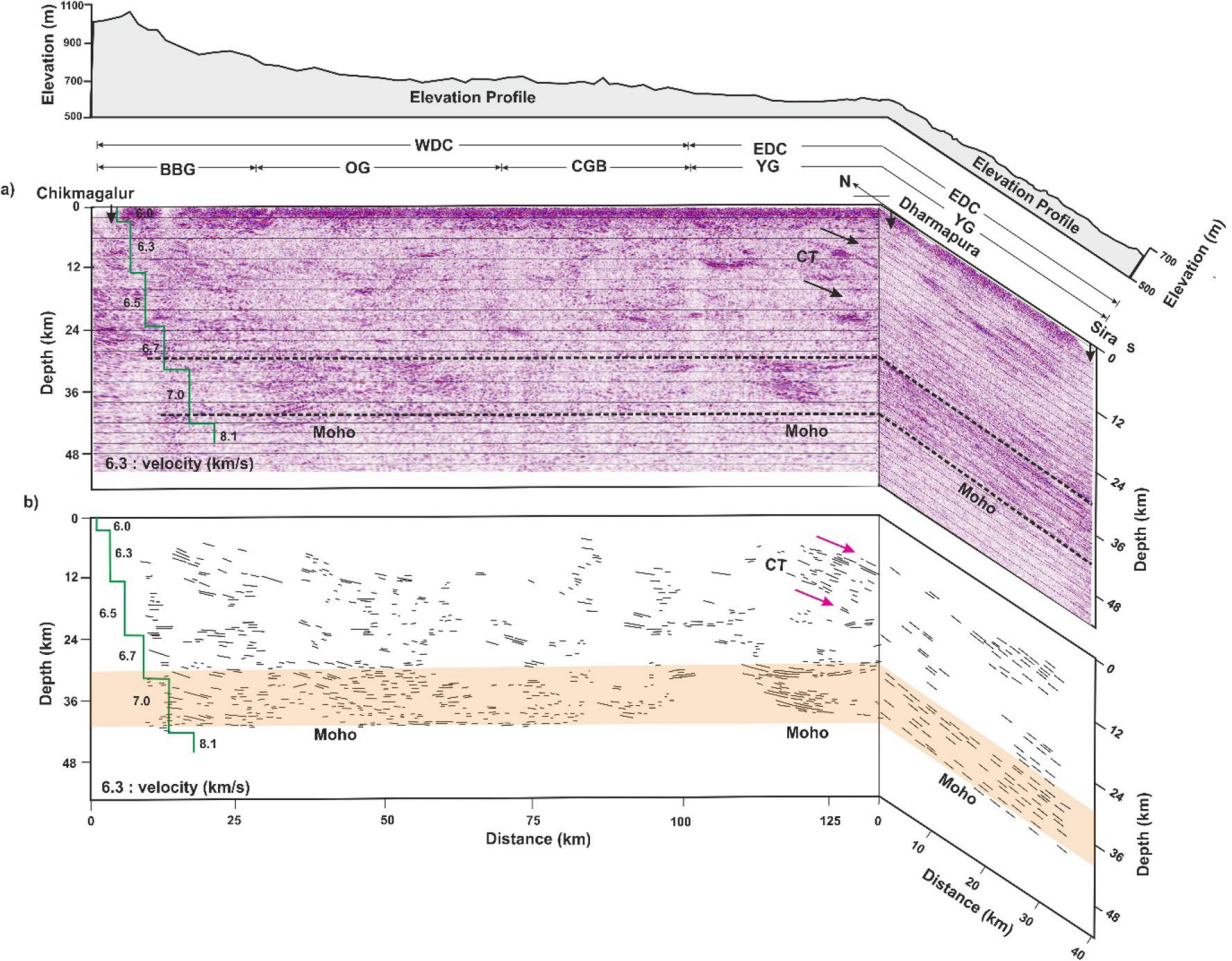
(**a**) Three-dimensional crustal seismic images along 130 km long Chikmagalur-Dharmapura, part of the (NE-SW) main profile and Dharmapura-Sira, the southern part of (N-S) cross-profile. The velocity-depth model of the main profile (Vijaya Rao et al.^11^) derived from the coincident refraction profile is marked over the seismic section. YG-Younger Granite; CGB-Chitradurga Greenstone Belt; CG-Closepet granite; EDC-Eastern Dharwar Craton; WDC-Western Dharwar Craton. The dashed lines from 30 to 40 km depth indicate the top and bottom of the subhorizontal reflection band observed in the lower-crust of the main and cross-profiles. The dashed line bottom indicates the Moho. (**b**) Line drawing showing prominent reflection bands from main and cross-profile. Elevation along both profiles are marked over the seismic sections.

A pseudo-3-D seismic section is prepared using the depth-migrated seismic images along the 130 km long Chikmagalur-Dharmapura segment of the main profile^21^ and the Dharmapura-Sira, the southern part of the present cross-profile. It is presented in Fig. 8. The 3-D crustal structure indicates that the Moho in this region is a nearly horizontal planar feature and acts as a structural detachment. The Moho in the region decouples the crust from the mantle as evidenced by the differences in the structure above and below it. Differences in rheological (mechanical) properties such as velocity, density, viscosity, and composition are responsible for the development of the detachment layer^11,21^. Deep crustal seismic reflection data from different parts of the world indicate that the lower crust or Moho acts as a regional detachment because of its ductile characteristics (Cook and Varsek^30^). Further, the subhorizontal lower-crustal reflection fabric is observed along the main-profile, extending to a length of 130 km to the west of Dharmapura (Figs. 2 and 8). It also covers a larger area along the cross-profile. Thus, we interpret the extensional activity observed here is a regional feature, which could not be inferred only with the earlier 2-D crustal structure.

**Figure 9:**
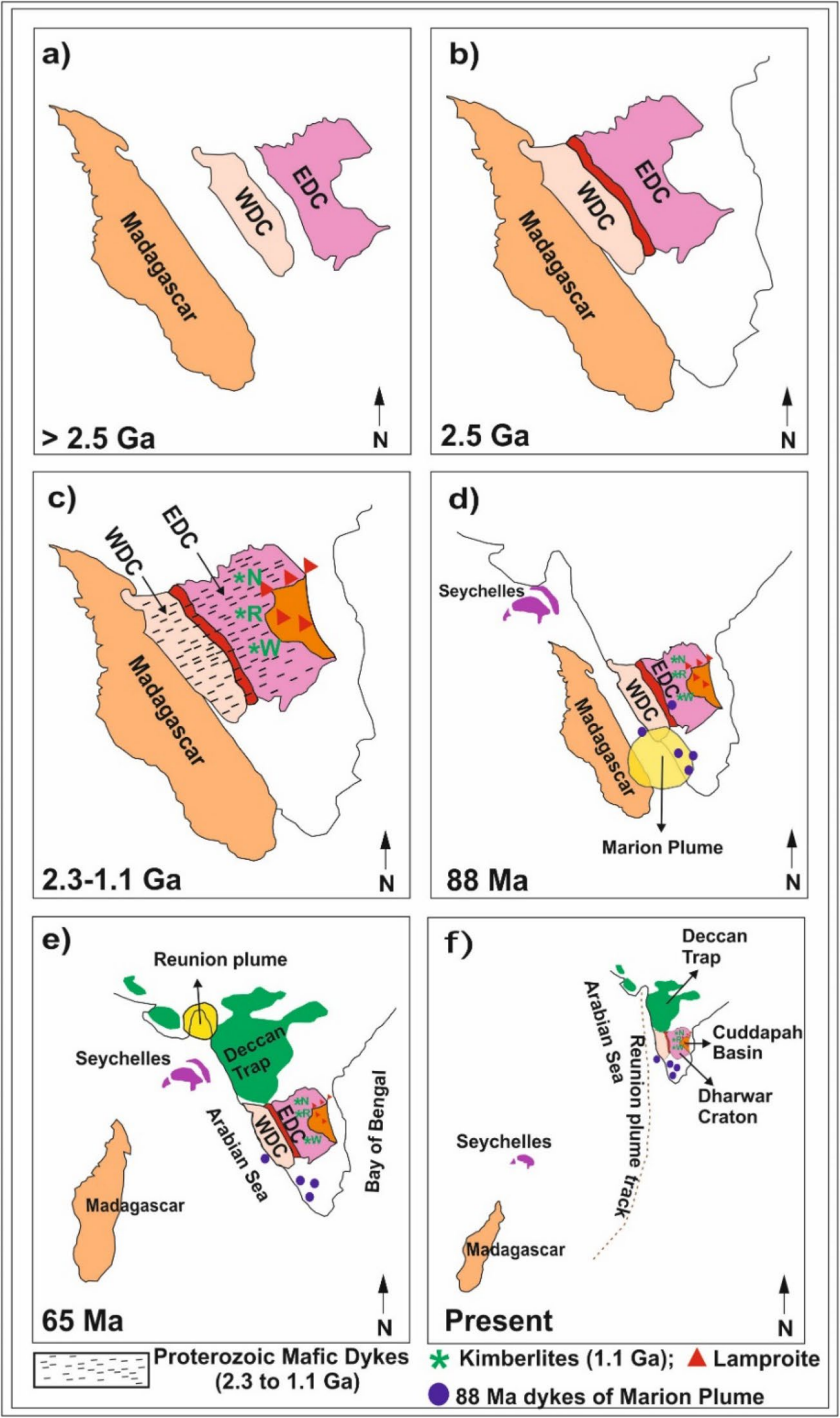
A cartoon illustrating the major tectonic activities on the west coast of the Indian shield. (**a**) Locations of Madagascar, western and eastern Dharwar cratons (WDC and EDC) earlier to 2.5 Ga, (**b**) Accretion of WDC and EDC during 2.5 Ga, (**c**) Mafic dyke swarms during the post-collisional period (2.3–1.1 Ga) and large-scale kimberlite-lamproite magmatic event at ca.1.2–1.1 Ga (**d**) Separation of Madagascar from the west coast of India due to the Marion plume activity at 88 Ma and opening of the ocean in the western part of Dharwar craton, (**e**) Separation of Seychelles from the west coast due to Reunion mantle plume activity at 65 Ma and emplacement of Deccan basalts, (**f**) Present-day locations of various units in south India, including the Dharwar craton. N-Narayanpet, R-Raichur, W-Wajrakarur are 1.1 Ga kimberlite locations.

The geodynamic evolution of the Dharwar craton is shown in the form of a schematic diagram in Fig. 9. The region experienced subduction-accretion activity between the WDC and EDC during ~ 2.5 Ga (Fig. 9b). The post-collisional extensional processes are observed in the form of Proterozoic (2.3–2.1 Ga) mafic dyke swarms (Fig. 9c)^31^, which might be responsible for the observed lower-crustal features up to some extent. During the post-collisional extensional process, the mantle material might have intruded into the lower crust and extruded laterally, producing flattening, stretching, and layering in the ductile lower-crust. Such an ordering process manifests as a subhorizontal reworked new Moho^25,32,33^. However, later tectonic/magmatic activities of the late Cretaceous and early Tertiary period played a significant role in evolving the lower-crustal and the Moho characteristics in the region, which are discussed below.

Breaking of the Gondwanaland during the Mesozoic^34^ is immensely affected the structure and tectonics of the Indian shield, especially the west coast. Madagascar was separated from the western part of India with the opening Arabian sea during ~ 88 Ma due to the Marion plume activity (Fig. 9d). This activity emplaced a large number of dykes on the west coast of India. Subsequently, Seychelles separated from India during ~ 65 Ma due to the Reunion mantle plume activities, which erupted wide-spread surface volcanism in the form of Deccan flood basalts (Fig. 9e). The Deccan volcanic province is one of the largest flood volcanic regions of the world. The Western Ghats (WG), with 1500 km long, paralleling the west coast and elevations greater than 1 km, is one of the largest escarpments on earth (Fig. 2). It might have formed as a rift shoulder during the rifting and breakup of Madagascar as indicated by the east-facing scarp in Madagascar is a mirror image of the west-facing scarp of the Western Ghats, India^7^. The Karnataka plateau located adjacent to the eastern part of WG (Fig. 2, KP) was also evolved during this process. It was reactivated during the separation of Seychelles from India. Some researchers suggest that the Western Ghats and the Deccan plateau, located to the north of the Karnataka plateau (Fig. 2, DP) were uplifted during the separation of Seychelles from India during 65 Ma. Even though the period of the uplift is debatable, it is certainly evolved between 88 and 65 Ma or the Karnataka and Deccan plateaus were uplifted respectively at ~ 88 Ma and ~ 65 Ma.

During the plume activity, a part of mantle material is accreted rheologically weak lower-crust. The seismic study identified a 10 km thick mantle material in the lower crust as a regional feature (Fig. 8). We interpret the major rifting/extensional activities related to the mantle plume episodes are responsible for the regionally extensive thick underplating in the lower-crust.

The presence of thick underplated material is constrained from several other geophysical studies. The high-velocity (7.1 km/s) lower-crustal layer (Fig. 8) derived from the coincident seismic refraction study is interpreted to represent magmatic underplating in the region^11,21^. Further, based on the identification of Seaward Dipping Reflectors (SDRs), Ajay et al.^35^ have identified the west coast of India as a volcanic rifted margin. Magmatic underplating along the volcanic margin is a common phenomenon. It might be responsible for the accretion of magma at the base of the crust, which is represented by high-velocity subhorizontal lower crustal fabric. Additionally, the shear wave velocity structure derived from receiver function analysis of earthquake data suggest a high-velocity lower-crustal layer representing magmatic underplating^36^. The thickness of the underplated layer is ~ 3 km, ~ 11 km, and 18 km beneath the EDC, WDC, and west-coast region (see Fig. 1 for locations). Further, 88 Ma leucogabbro dyke swarms observed on the west coast, St Mary islands, as well as in the interior of the region^37^ (Fig. 8d) are the manifestation of magmatic underplating in the region and related to the Marion plume activity. The above geophysical evidence complements the underplating identified from the present study.

When mafic melt from the mantle with a velocity of 8.0–8.3 km/s and density of 3.3 g/cm^3^ intrudes into the ductile lower crust gets mixed up with the already existing felsic/intermediate crustal material. Such a lower crustal accretionary process is referred to magmatic underplating. Now, the lower crust exhibits higher velocity and density respectively of the order of 7.0–7.4 km/s and 2.9–3.1 g/cm^3^ compared with earlier values. Such an additional regionally extending subsurface load disturbs the isostatic balance, which will be compensated by the surface uplift. Thus, we interpret, the magmatic underplating identified here generated isostatic uplift and responsible for the epeirogenic uplift in the region. Radhakrishna et al.^42^ suggest igneous underplating is responsible for the plateau uplift in the region. Mantle plume/hotspot related uplift is a major tectonic process that covers 10% of the earth’s surface. Many continental uplifts are associated with basaltic volcanism^43^. The width of the uplift can vary from 500 to 1000 km and 1–3 km high, as observed from several parts of the globe.

Magmatic underplating is considered as a possible mechanism at several places, e.g., for the regional uplift of the Colorado Plateau^45^, the western margin of the Yangtze craton China^46^. The intrusion of the great thickness of magma into the lower-crust is generally associated with uplift, especially non-plate boundary/intraplate regions, like the Karnataka plateau^1,4,47^. McKenzie^4^ suggests the addition of 15 km of mantle material to the lower-crust may produce 2.7 km of uplift, depending on the densities of the mantle and the accreted material. The present study, constrained from other geophysical and geological data, suggests a relationship between extension/rifting, volcanism, and uplift.

The relationship between magmatic underplating and the corresponding expected elevation due to isostatic processes is provided by a simple formula^48^

$$\Delta {\text{h }} = \, \Delta {\text{r }}(\rho_{{\text{m}}} - \rho_{{\text{r}}} ) \, / \, \rho_{{\text{h}}}$$

where, Δh is excess elevation, Δr is the thickness of the underplated layer, ρ_m_ is the density of mantle, ρ_r_ density of underplated layer, and ρ_h_ density of elevated portion. The thickness of the underplated layer (Δr) derived from seismic images is 10 km. The densities of the upper mantle (ρ_m_), underplated layer (ρ_r_), and the elevated portion (ρ_h_) are 3.31 g/cm^3^, 2.97 g/cm^3^, and 2.69 g/cm^3^ respectively. They are taken from the density model derived from the velocity-depth model derived from refraction data, which were acquired along the present reflection profile^11^. Substituting these values in the above equation gives

$$\Delta {\text{h}} = \, 1.26 \, \;{\text{km}}.$$

The residual (excess) topography is estimated by the difference between the expected (paleo, 1260 m) and actual (present, 600 m) elevation, which is of the order of 700 m. We interpret the discrepancy is due to the flexural response to combined onshore denudational unloading and offshore sediment loading (Fig. 3). It is constrained from the studies by Campanile et al.^7^ and Richards et al.^5^, who suggested a high rate of denudation and clastic sediment loading in the offshore basins during the Cenozoic is compensated due to flexural isostasy.

Variation of elevation according to the density of the underplated layer (ρ_r_) for a constant thickness of the underplated layer and mantle density is given below.

$${\text{Uplift}}, \, \Delta {\text{h}} = { 1}.{\text{71 km}} \, {\text{for }}\rho_{{\text{r}}} = { 2}.{\text{85 g}}/{\text{cm}}^{{3}} ,$$

$$\Delta {\text{h}} = { 1}.{\text{52 km}} \, {\text{for }}\rho_{{\text{r}}} = { 2}.{9}0{\text{ g}}/{\text{cm}}^{{3}} ,$$

$$\Delta {\text{h}} = { 1}.{\text{15 km}} \, {\text{for }}\rho_{{\text{r}}} = { 3}.00{\text{ g}}/{\text{cm}}^{{3}} ,$$

$$\Delta {\text{h}} = \, 0.{\text{44 km}} \, {\text{for }}\rho_{{\text{r}}} = { 3}.{\text{19 g}}/{\text{cm}}^{{3}} .$$

Erosion is a natural process which contributes to the epeirogeny of a region. The erosional rate in the region is not constant throughout. A maximum of 4–5 km of denudation is observed in the last 150 Ma, which amounts to 26–33 m/Myr^49^. In another study using modelled thermal histories of the apatite fission track dates suggest higher rates of denudation at the beginning of Cenozoic with an increased erosion in the middle of Eocene. That data suggest3–4 km of denudation close to the coast and 1.5–2.5 km inside the continental region, which is constrained by 4 km thick sediments in the offshore Konkan-Kerala basin^50^. Numerical modelling and mass balance studies of flexural responses to onshore denudational unloading and offshore sediment loading by Richards et al.^5^ and Campanile et al.^51^ suggest flexural isostasy alone can’t produce a significant amount of offshore sediment deposition and requires a pre-existing elevated plateau portion. The additional paleo-elevation required at the onset of denudation is provided by the magmatic underplating imaged in the present study.

Plume activity may cause initial surface uplift, but the geological and geomorphological data suggest the uplift continues long after the plume effects have decayed^6^. Radhakrishna^9^ suggests constructive uplift and destructive erosion are a continuous process and shaping the peninsular Indian landscape since Neogene. We opine that the longevity of the uplift from 88 Ma to the present (Figs. 2 and 8, Elevation profile) can be better explained by denudational isostasy (Fig. 3), which provides a long-term mechanism for the continuing process of uplift.

Tappe et al.^38^ and Shaikh et al.^39^ from the kimberlite studies on the Dharwar craton provided convincing evidence for the existence of a relatively thick lithosphere (~ 190 km) till 1.1 Ga. Subsequently, the mantle lithosphere was delaminated, leading to a thinner lithosphere (~ 120 km). Major post-1.1 Ga tectonic activity experienced by the Dharwar craton is eparation of India from the Gondwana/Pangea supercontinent during the Mesozoic. After separating from the Gondwana supercontinent, the Indian plate drifted to the north, covering a distance of ~ 7500 km with a speed of 15–20 cm/year, and collided with Eurasia forming the Himalayas at ~ 55 Ma^8^. This unique episode along with high mantle heat flux derived from the Marion and Reunion mantle plume activities, might have reduced the lithospheric thickness to ~ 110 km beneath the Dharwar craton^40,41^. It could be possible that lithospheric thinning as observed in the region might also be expected to be present a mechanism for causing uplift in addition to the magmatic underplating. The response to the gravitational imbalance due to these activities generated isostatic uplift and formation of the plateau.

Raimondo et al.^52^ suggest plate-boundary stresses are transmitted over a large distance (> 1000 km) through the lithosphere, which acts as an effective stress guide. These stresses can control the tectonic evolution of the continental interior. Peninsular India is in a state of compression between the Himalayan collision zone in the NE and the Indian ocean ridge push^53^ in the SW. Thus, we opine that the periodic uplifts may be a consequence of isostatic adjustments due to the collision of India with Eurasia (~ 55 Ma) or the slowdown in plate velocity due to this collision. It could also be due to the onset of the Indian monsoon during 15–8 Ma, which has some effect on erosion rate and modern-day uplift. Thus, we suggest that the continuation of erosion processes will lead to further exhumation, associated isostatic uplift and seismicity in the region. The plateau uplift in the region is a continuous process with flexural adjustment and could be responsible for the neotectonics activity as suggested by Valdiya^44^.

Thermally driven models, such as active rifting triggered by mantle plumes, predict plateau uplift, but the uplift is transient due to expected thermal and convective decay with time. They can’t explain the long-lived uplift experienced in the Karnataka plateau and other passive margins^6^. Normal upper mantle velocity beneath the plateau^11^ suggests the absence of a hotter mantle, and no thermal expansion is expected. These velocities are compatible with the normal heat flow values ranging between 25 to 50 mW/m^2^ with a mean value of 36 mW/m^2^, similar to many Archean terrains^54^. The Bouguer gravity values are low and vary between − 120 and − 70 mGals over the Karnataka plateau^11,55^ and are consistent with the crustal thickening. There is a positive relationship between elevation and crustal thickness, indicating the region is isostatically compensated. Airy (local) isostasy is an end-member of flexural isostasy. The entire lithosphere of peninsular India is in a state of isostatic equilibrium and that the variation of loads is entirely supported by the strength of the lithosphere^8,56^.

## Conclusions

The pseudo-3-D crustal structure derived from orthogonal profiles identified 10 km thick subhorizontal lower-crustal fabric associated with a high-velocity (7.1 km/s) layer which is interpreted as magmatic underplating. A consequence of this process is the generation of an equilibrated younger Moho. It might have formed during the extensional/rifting process in the region. The extensional activity is identified as a regional feature based on the coverage of lower-crustal fabric to a large area both along and across the strike and on other geophysical data. Rifting and separation of the Madagascar and Seychelles from India due to the Marion and Reunion mantle plume activities during 88 Ma and 65 Ma are responsible for the wide-spread underplating, which in turn responsible for the epeirogenic uplift and formation of the Karnataka plateau. Onshore denudational unloading and offshore sediment loading and associated denudational/flexural isostasy is another important factor responsible for the plateau uplift in the region. The causes for uplift covering a vast area with different geological features are multi-genetic. We believe a single unifying explanation for the uplift may be difficult at this stage.

The present study is global in nature that suggests a relationship between the mantle plumes, rifting (extension), development of continental margins, plateau uplift, and denudational isostasy. The model presented here for the evolution and persistence of elevated Indian topography may be applicable to other escarpments on the earth.

## Data Availability

The datasets generated during and/or analysed during the current study are available from the corresponding author on reasonable request.
